# Trajectories of Mother–Child Closeness and Child Behavioural and Emotional Outcomes in Families of Children With Intellectual Disabilities

**DOI:** 10.1111/jir.70110

**Published:** 2026-05-08

**Authors:** Emma L. Taylor, Paul A. Thompson, Samantha Flynn, Kylie M. Gray, Richard P. Hastings

**Affiliations:** ^1^ Intellectual Disabilities Research Institute (IDRIS), School of Social Policy and Society University of Birmingham Edgbaston UK; ^2^ Centre for Research in Intellectual and Developmental Disabilities University of Warwick Coventry UK; ^3^ Department of Psychiatry, School of Clinical Sciences at Monash Health Monash University Melbourne Australia

**Keywords:** behavioural problems, intellectual disability, mother–child closeness, prosocial behaviour, trajectories

## Abstract

**Background:**

Children with intellectual disabilities display fewer prosocial behaviours and increased behavioural and emotional problems compared to children without intellectual disabilities. Mother–child closeness may be an important factor in improving behavioural and emotional outcomes in children with intellectual disabilities over time. We aimed to examine the covarying relationship between mother–child closeness and child externalising and internalising behaviour problems, and child prosocial behaviour, respectively, over time.

**Methods:**

Parallel process growth modelling was conducted using data from 353 maternal primary caregivers who took part in three waves of the 1000 Families Study. Mother–child closeness was measured at each wave using the Child–Parent Relationship Scale. Child behavioural and emotional outcomes were measured using the Strengths and Difficulties Questionnaire. We controlled for time‐varying and time‐invariant covariates including the child's level of communication skills, autism diagnosis, maternal psychological distress and family economic adversity.

**Results:**

The trajectory of mother–child closeness remained relatively stable across the three waves. After controlling for covariates, the trajectories of mother–child closeness and child prosocial behaviour significantly covaried. However, the trajectory of mother–child closeness did not significantly covary with the trajectory of either child internalising or externalising behaviour problems.

**Conclusion:**

Interventions aiming to improve child prosocial behaviour and/or mother–child closeness in families of children with intellectual disabilities may benefit from considering the relationship between mother–child closeness and child prosocial behaviour. Findings highlight the need for additional research to understand the relationship between these factors and further examine underlying individual differences in these families.

## 1 Introduction

Children with intellectual disabilities typically display increased rates of behaviour and emotional problems and fewer prosocial behaviours compared to typically developing children (Bailey et al. 2019; Buckley et al. 2020; Kaptein et al. 2008). Longitudinal studies have shown that these higher rates of problem behaviours are evident in early to middle childhood (Bailey et al. 2019; Emerson and Einfeld 2010) and continue into adulthood with some relative improvement (De Ruiter et al. 2007; Einfeld et al. 2006). For families of children with intellectual disabilities, increased child problem behaviours can lead to reduced maternal well‐being (Blacher and Baker 2019; Neece et al. 2012; Williams et al. 2022) and overall family functioning (Baker et al. 2003), leading to greater care costs (Einfeld et al. 2010). Therefore, identifying factors that positively influence children's behavioural and emotional outcomes may be important in these families.

Theories of child development posit the importance of the parent–child relationship in shaping children's behavioural and socioemotional outcomes. According to attachment theory, a warm, close and secure parent–child relationship is fundamental to the development of children's internal working models, which influence how they perceive the world, themselves and others, as well as their ability to navigate and overcome stressful and/or adverse situations (Bowlby 1977). Children who have an insecure or less close relationship with their parents are at an increased risk of later behavioural and emotional problems, including anxiety and depression (Bowlby 1977). Extending this perspective, Bronfenbrenner's bioecological model describes the parent–child relationship and child behavioural and socioemotional outcomes as reciprocally influencing one another, with this proximal process shaped by child and parental characteristics and broader contextual factors, including the familial environment (Bronfenbrenner and Ceci 1994). As such, examining the reciprocal association between the parent–child relationship and children's behaviour may offer further insights into children's developmental outcomes.

However, empirical research has typically examined the unidirectional association between the parent–child relationship and children's behavioural and emotional outcomes in families of children with intellectual disabilities. Reduced parent–child closeness has been associated with greater child behavioural problems (Schuiringa et al. 2015; Teague et al. 2020; Totsika et al. 2014) and fewer child prosocial behaviours (Williams et al. 2025). This finding similarly reflects previous research findings in the typically developing literature (e.g., Dreidi et al. 2024; Katsantonis and McLellan 2023). However, mothers of children with intellectual disabilities have reported a less close relationship to their child compared to mothers of typically developing children (Aksoy and Kobya Bulut 2025; Totsika et al. 2014), which could be bidirectionally associated with increases in child behaviour and emotional problems and fewer child prosocial behaviours (Bronfenbrenner and Ceci 1994). Exploring how mother–child closeness and child behavioural and emotional outcomes may influence one another over time could help inform targeted intervention support to improve family outcomes in families of children with intellectual disabilities.

Although longitudinal research has examined children's behavioural and emotional outcomes in families of children with intellectual disabilities (e.g., Bailey et al. 2019; De Ruiter et al. 2007; Emerson and Einfeld 2010), relatively little is known about how mother–child closeness develops over time in these families. In families of children with Fragile X syndrome, Fielding‐Gebhardt et al. (2020) reported that the trajectory of mother–child closeness remained relatively stable over time. Conversely, in families of typically developing children, findings have generally suggested that mother–child closeness or warmth declines between early or middle childhood and early adolescence (Nomaguchi and Allen 2023; Shanahan et al. 2007; Xie et al. 2021; Yan et al. 2019).

To the best of the authors' knowledge, no research to date has examined the reciprocal relationship between mother–child closeness and child behavioural and emotional outcomes over time in families of children with intellectual disabilities. Examining whether and how mother–child closeness and child behaviour covary over time allows us to consider whether there is a bidirectional relationship in the development of these factors over time, rather than examining for time‐specific directional associations. The current study therefore aimed to address the following research question: How does the trajectory of mother–child closeness covary with the trajectory of child behaviour (externalising behaviour problems, internalising behaviour problems and prosocial behaviour)? We hypothesised that increases in mother–child closeness would be associated with fewer child externalising and internalising behaviour problems, respectively, and increases in child prosocial behaviour over time. Conversely, we hypothesised that increases in child externalising and internalising behaviour problems would be associated with reduced mother–child closeness, whereas increases in child prosocial behaviour would be associated with increases in mother–child closeness over time.

In line with Bronfenbrenner's bioecological model, we controlled for child, mother and environmental factors that may influence a covarying relationship between mother–child closeness and child behaviour over time. These included an additional diagnosis of autism, maternal psychological distress, child's level of communication skills and family economic adversity, which have been associated with closeness and/or child behavioural and emotional outcomes in families of children with intellectual disabilities (Bailey et al. 2019; Chadwick et al. 2008; Emerson and Hatton 2007; Hastings et al. 2006; Howe 2006; Neece et al. 2012; Sterkenburg et al. 2022; Teague et al. 2020; Totsika et al. 2020, 2011; Zabidi et al. 2023).

## 2 Methods

### 2.1 Study Design

We analysed data using the 1000 Families Study (Hastings et al. 2020). The 1000 Families Study is a large UK longitudinal cohort study involving 1184 families of children with intellectual disabilities aged between 4 and 15 years and 11 months at Wave 1 (Hastings et al. 2020). The current study drew upon data collected at Wave 1 (November 2015 to January 2017), Wave 2 (August 2018 to August 2021) and Wave 3 (December 2021 to January 2023).

### 2.2 Participants

Data from 509 primary caregivers who took part in all three waves of the 1000 Families Study were included in the analysis (Wave 1 = 1184; Wave 2 = 650; Wave 3 = 577). Informed consent was sought from families at each wave (see Hastings et al. 2020). Families were excluded from the current study if participants did not identify as a maternal primary caregiver at any given wave (*n* = 20) and if their child with intellectual disabilities was over 16 years of age at Wave 3 (*n* = 136), as the survey completed by these families did not measure child behaviour. A final sample of 353 maternal primary caregivers was included in the analysis.

At Wave 1, most participants identified as the child's biological mother (93.47%) and were White British (87.82%). Nearly 70% of children with intellectual disabilities were male (69.1%). Mean child ages at each wave were 7.33 years (SD = 1.91 years), 10.31 years (SD = 1.98 years) and 12.94 years (SD = 1.92 years). Mothers reported that 236 of the children with intellectual disabilities were also autistic. Further information on the primary caregiver and family demographic information can be found in Table 1.

**TABLE 1:** Primary caregiver and family demographic information at Wave 1 (*N* = 353).

Variable	*n* (%)
Relationship to the child	
Biological mother	330 (93.47%)
Adoptive mother	14 (3.97%)
Grandmother	8 (2.27%)
Foster mother	1 (0.28%)
Ethnicity	
White British	310 (87.82%)
White (Irish/Travelling Community/Other)	21 (5.95%)
Asian/Asian British (Indian/Pakistani/Bangladeshi/Chinese/Other)	9 (2.55%)
Multiple/mixed ethnic groups	7 (1.97%)
Black (African/Caribbean/Black British/Other)	3 (0.85%)
Missing data	3 (0.85%)
Employment	
In a job (employed/self‐employed)	164 (46.46%)
Looking after family and home	138 (39.09%)
Other (full‐time student/voluntary work/do something else)	46 (13.03%)
Unemployed	4 (1.13%)
Missing data	1 (0.28%)
Education	
Degree (e.g., BA, BSc and MA)	185 (52.41%)
Higher education but below degree level	78 (22.10%)
A/AS levels/GCSEs or equivalent	77 (21.81%)
No qualifications	1 (0.28%)
Missing data	12 (3.4%)
Marital status	
Married	224 (63.46%)
Living with partner	57 (16.15%)
Single/divorced/separated/widowed/not currently living with partner	56 (15.86%)
Missing data	16 (4.53%)
UK median weekly income	
Below £700	221 (62.6%)
Above £700	115 (32.58%)
Missing data	17 (4.82%)

### 2.3 Measures

#### 2.3.1 Mother–Child Closeness

Mother–child closeness was measured at all three waves using the closeness subscale of the Child–Parent Relationship Scale‐Short Form (CPRS‐SF; Pianta 1995). Participants were asked to respond to seven items (e.g., ‘I share an affectionate relationship with this child’) using a 5‐point Likert‐type scale (1 = *definitely does not apply* to 5 = *definitely applies*). Scores are summed to calculate a total perceived closeness score, with a higher total score indicative of greater perceived mother–child closeness.

Psychometric properties of the CPRS‐SF closeness subscale are reported to have satisfactory internal consistency and construct validity, as measured using maternal‐reported data in a sample of young children without intellectual disabilities (Driscoll and Pianta 2011). Evidence of its validity has also been reported when examining the parent–child relationship quality in families of non‐disabled children using cross‐cultural contexts (Escalante‐Barrios et al. 2020). Although the validity of this measure has not been tested in families of children with intellectual disabilities, the CPRS‐SF has demonstrated acceptable Cronbach's alpha in studies examining parent–child closeness in families of children with intellectual disabilities (e.g., Totsika et al. 2020; Zabidi et al. 2023). In the current study, the CPRS‐SF closeness subscale showed acceptable reliability at Wave 1 (*ω* = 0.78).

#### 2.3.2 Child Behaviour

Child internalising and externalising behaviour problems and child prosocial behaviour were measured at all three waves using the Strengths and Difficulties Questionnaire (SDQ; Goodman 1997). Participants were asked to respond to 25 items based on their child's behaviour in the last 6 months using a 3‐point scale (0 = *not true* to 2 = *certainly true*). Child prosocial behaviour was measured using five items (e.g., ‘Considerate of other people's feelings’). Child internalising behaviour problems were measured by summing the scores from the emotional problems subscale (five items; e.g., ‘Many worries, often seems worried’) and the peer relationship problems subscale (five items; e.g., ‘Rather solitary, tends to play along’). Child externalising behaviour problems were measured by combining scores from the conduct behaviour subscale (five items; e.g., ‘Often loses temper’) and the hyperactivity/inattention subscale (five items; e.g., ‘Restless, overactive, cannot stay still for long’).

The SDQ has satisfactory reliability in measuring behavioural and emotional outcomes in typically developing children (Goodman 2001) as well as good concurrent validity (Muris et al. 2003). Further, as measured using a sample of 626 children and adolescents with intellectual disabilities, the SDQ is clinically useful in identifying behaviour and emotional problems in this population (Murray et al. 2021). In the current study, all subscales had reasonably good McDonald's omega reliability at Wave 1 (externalising: *ω* **=** 0.69; internalising: *ω* **=** 0.74; prosocial: *ω* **=** 0.82).

### 2.4 Time‐Varying Covariates

#### 2.4.1 Maternal Psychological Distress

Maternal psychological distress was measured at each wave using the Kessler 6 (K6; Kessler et al. 2002). Participants were asked to rate how often they experienced six symptoms (e.g., ‘hopeless?’ and ‘nervous?’) in the last 30 days. The six items are rated using a 5‐point Likert scale (0 = *none of the time* to 4 = *all of the time*) and summed to calculate a total score. Higher total scores indicate greater levels of maternal psychological distress.

The K6 is recognised as a valid scale for assessing clinically concerning levels of psychological distress in families of children without intellectual disabilities (Kessler et al. 2010; Prochaska et al. 2012). Although the validity of the K6 has not been tested in families of children with intellectual disabilities, it has shown good reliability in previous research studying these families (e.g., Grey et al. 2018; Totsika et al. 2011; Sutherland et al. 2024; Williams et al. 2025). In this study, the K6 demonstrated good omega reliability at Wave 1 (*ω* **=** 0.88).

#### 2.4.2 Family Economic Adversity

Family economic adversity was measured at all three waves using participant responses to two questions: ‘How well would you say you're managing financially these days?’ and ‘How hard would it be to raise £2000 in an emergency given one week's notice?’. The first question was scored using a 5‐point scale (1 = *living comfortably?* to 5 = *finding it very difficult?*), with the latter scored using a 4‐point scale (1 = *I could easily raise the money* to 4 = *I do not think I could raise the money*).

The items used in the current study have been included in part in previous research drawing upon data from the 1000 Families Study dataset (e.g., Williams et al. 2022, 2023, 2025) and other large secondary datasets such as the Millennium Cohort Study (e.g., Bailey et al. 2021; Hayden et al. 2019; Hayden et al. 2023). Following the methods applied in these previous studies, total composite scores at each wave were obtained by summing item scores; a higher total score is indicative of the family experiencing greater economic adversity. In the current study, the items had good internal consistency at Wave 1 (*ω* **=** 0.71).

### 2.5 Time‐Invariant Covariates

#### 2.5.1 Child's Level of Communication Skills

Participants reported their child with intellectual disabilities' level of everyday communication skills at Waves 2 and 3 using three items. Two of these items derived from the communication subscale of the GO4KIDDS Brief Adaptive Scale (Perry et al. 2015). A further third item in the same format was added by the 1000 Families research team to measure the child's use of alternative methods of communication (e.g., signing, symbol gestures and Makaton). All three items were scored on a 5‐point scale, ranging between 1 (indicating lower levels of communication skills) and 5 (indicating greater communication skills). A total communication skills score was calculated by combining the score for the child's receptive communication skills and the highest score for either the child's spoken communication skills or communication skills using alternative methods of communication; a higher total score is indicative of better communication skills. If data were available at Waves 2 and 3, data from the wave with the highest total score were included in the analysis to accurately represent the child's highest level of communication skills.

The GO4KIDDS Brief Adaptive Scale is recognised as a reliable and valid measure of adaptive behaviours (including communication skills) in children with intellectual disabilities (Perry et al. 2015). All three items used in the current study as a measure of the child's communication skills have been used in previous research concerning children with intellectual disabilities (e.g., Bailey et al. 2021).

#### 2.5.2 Child's Additional Diagnosis of Autism

At all three waves, participants reported whether their child had received a diagnosis of autism, in addition to intellectual disabilities. A child with intellectual disabilities was included in the analysis as being autistic if it was reported by the participant at any wave.

### 2.6 Statistical Analysis

Before any data were accessed, this study was pre‐registered on Open Science Framework (https://osf.io/8pumf). All analyses were conducted using the statistical software, R (version 4.3.2), and the *lavaan* package (Rosseel 2012).

Parallel process growth curve modelling fitted within the Structural Equation Modelling (SEM) framework was used to address the aims of this study (Curran et al. 2010). First, univariate growth curve trajectory models were plotted to examine the independent trajectory development of perceived mother–child closeness and each child behaviour outcome (externalising behaviour problems, internalising behaviour problems and prosocial behaviour; Bollen and Curran 2006). As there were three waves of data, the models were limited to the identification of linear trajectories. Univariate model fit statistics were assessed for the goodness‐of‐fit to the observed data as well as the statistical significance and direction of growth parameters.

Next, three unconditional linear parallel growth curve models were fitted to explore how the trajectory of mother–child closeness co‐developed with the trajectory of each child behaviour outcome. For each model, model fit statistics were examined to determine the model's goodness‐of‐fit and also the statistical significance of growth parameters and their reciprocal effects. Finally, three conditional parallel growth curve models were fitted to test whether any covariates contributed to the trajectory growth of each outcome variable. We deviated from the study pre‐registration when measuring family economic adversity, as the calculated UK median score for income poverty originally planned for inclusion in the composite variable was not available at Wave 2.

Model fit statistics were assessed using Tucker–Lewis index (TLI > 0.90), comparative fit index (CFI > 0.90), root mean square error of approximation (RMSEA < 0.08) and standardised root mean square residual (SRMR < 0.08) (Hu and Bentler 1999; Wang and Wang 2019). Ninety per cent confidence intervals (CIs) were examined when assessing RMSEA (Wang and Wang 2019). Additionally, the *p*‐values reported in the chi‐squared output were examined using the standard *p* < 0.05 to determine the statistical significance of the model fit.

The amount of missingness ranged between 0% (child age at Waves 1 and 3; child diagnosis of autism) and 5.67% (mother–child closeness at Wave 1) across the three waves. Data were considered missing completely at random (MCAR; Arbuckle 2013; Little 1988); however, all growth models were fitted using full information maximum likelihood estimation for completeness, but it is unlikely that any substantial bias was related to missingness, given the low proportion missing.

## 3 Results

Table 2 presents the means, standard deviations and omega coefficients for mother–child closeness and child behaviour (externalising behaviour problems, internalising behaviour problems and prosocial behaviour) and the following covariates: maternal psychological distress, family economic adversity and child's level of communication skills.

**TABLE 2:** Mean, standard deviation and McDonald's omega for outcome measures, time‐varying covariates and time‐invariant covariate.

Variable	Wave 1			Wave 2			Wave 3		
	Mean	SD	*ω*	Mean	SD	*ω*	Mean	SD	*ω*
Outcomes									
Mother–child closeness	26.21	4.95	0.78	26.83	5.20	0.77	26.74	5.43	0.80
Child prosocial behaviour	3.83	2.75	0.82	4.21	2.83	0.83	4.33	2.81	0.84
Child internalising behaviour problems	9.40	4.07	0.74	9.90	4.05	0.72	10.00	4.03	0.74
Child externalising behaviour problems	11.50	3.09	0.69	11.29	3.36	0.72	10.64	3.43	0.72
Time‐varying covariates									
Maternal psychological distress	8.90	5.49	0.88	8.76	5.26	0.87	9.07	5.71	0.90
Family economic adversity	4.88	1.83	0.71	4.83	1.92	0.73	4.86	1.95	0.78
Time‐invariant covariate									
Child's communication level				6.43	2.32	0.71	6.77	2.35	0.86

Before fitting the conditional models (i.e., those including all covariates), we first established whether an unconditional linear growth model in each outcome measure and unconditional parallel process models for pairs of outcomes provided sufficient fit to the data. All provisional models showed suitable model fit across all outcomes (Tables S1–S9). Following pre‐registered steps, we report the relation between paired trajectories in the unconditional parallel process models and the parameter estimates in each outcome from these models.

### 3.1 Unconditional Parallel Process Growth Models

#### 3.1.1 Covariation Between Mother–Child Closeness and Child Externalising Behaviour Problems

As shown in Table 3, although there were no significant average rates of change over time in either outcome, the trajectories of mother–child closeness and child externalising behaviour problems negatively covaried at Wave 1 and over time (intercept: *b* = −3.779, SE = 0.825, *p* < 0.001; slope: *b* = −0.401, SE = 0.165, *p* = 0.015), suggesting that increases in mother–child closeness were associated with fewer child externalising behaviour problems over time (and vice versa). Significant variation was present in both outcomes at Wave 1 (closeness: *b* = 18.365, SE = 2.131, *p* < 0.001; externalising: *b* = 6.918, SE = 0.894, *p* < 0.001) and in mother–child closeness over time (*b* = 1.737, SE = 0.877, *p* = 0.048), indicating the presence of individual‐level change in mother–child closeness over time.

**TABLE 3:** Parameter estimates for unconditional parallel process growth modelling of trajectory of mother–child closeness and trajectory of child externalising behaviour problems.

	Coefficient	Std. error	CI (95%)		*p*
			Lower	Upper	
Intercepts					
iCloseness	26.302	0.263	25.786	26.819	< 0.001***
iExternal	11.572	0.164	11.250	11.894	< 0.001***
sCloseness	−0.369	2.120	−4.524	3.785	0.862
sExternal	−0.780	1.370	−3.465	1.905	0.569
Regressions					
sExternal ~ iCloseness	0.007	0.025	−0.042	0.057	0.773
sExternal ~ iExternal	0.014	0.073	−0.129	0.158	0.845
sCloseness ~ iExternal	0.082	0.063	−0.042	0.206	0.196
sCloseness ~ iCloseness	−0.011	0.061	−0.131	0.109	0.852
Variances					
iCloseness	18.365	2.131	14.189	22.541	< 0.001***
sCloseness	1.737	0.877	0.019	3.455	0.048*
iExternal	6.918	0.894	5.166	8.670	< 0.001***
sExternal	0.319	0.397	−0.460	1.098	0.422
Covariances					
iCloseness ~~ iExternal	−3.779	0.825	−5.397	−2.162	< 0.001***
sCloseness ~~ sExternal	−0.401	0.165	−0.724	−0.077	0.015*

Abbreviations: CI = confidence interval; Closeness = mother–child closeness; External = child externalising behaviour problems; i = intercept factor; s = slope factor.

*** *p* < 0.001.

* *p* < 0.05.

#### 3.1.2 Covariation Between Mother–Child Closeness and Child Internalising Behaviour Problems

Similar to the previous model, the trajectories of mother–child closeness and child internalising behaviour problems remained stable over time. As displayed in Table 4, mother–child closeness and child internalising behaviour problems negatively covaried at Wave 1 (*b* = −4.115, SE = 1.078, *p* < 0.001). Thus, children with a close relationship with their mother were more likely to display fewer child internalising behaviour problems at Wave 1 (and vice versa). However, this covarying relationship did not continue over time. Significant variation in the trajectories of mother–child closeness and child internalising behaviour problems was present at Wave 1 (closeness: *b* = 19.465, SE = 2.252, *p* < 0.001; internalising: *b* = 13.586, SE = 1.461, *p* < 0.001) and over time (closeness: *b* = 2.410, SE = 0.812, *p* = 0.003; internalising: *b* = 1.055, SE = 0.444, *p* = 0.017). Therefore, this finding highlights individual differences in these outcomes over time.

**TABLE 4:** Parameter estimates for unconditional parallel process growth modelling of trajectory of mother–child closeness and trajectory of child internalising behaviour problems.

	Coefficient	Std. error	CI (95%)		*p*
			Lower	Upper	
Intercepts					
iCloseness	26.249	0.263	25.733	26.765	< 0.001***
iInternal	9.491	0.217	9.066	9.917	< 0.001***
sCloseness	1.621	1.561	−1.439	4.681	0.299
sInternal	0.899	0.890	−0.846	2.643	0.313
Regressions					
sInternal ~ iCloseness	0.014	0.023	−0.032	0.060	0.540
sInternal ~ iInternal	−0.104	0.045	−0.191	−0.017	0.020*
sCloseness ~ iInternal	0.021	0.038	−0.054	0.095	0.583
sCloseness ~ iCloseness	−0.059	0.052	−0.160	0.043	0.260
Variances					
iCloseness	19.465	2.252	15.051	23.880	< 0.001***
sCloseness	2.410	0.812	0.817	4.002	0.003**
iInternal	13.586	1.461	10.722	16.449	< 0.001***
sInternal	1.055	0.444	0.185	1.924	0.017*
Covariances					
iCloseness ~~ iInternal	−4.115	1.078	−6.228	−2.001	< 0.001***
sCloseness ~~ sInternal	0.125	0.182	−0.231	0.482	0.490

Abbreviations: CI = confidence interval; Closeness = mother–child closeness; i = intercept factor; Internal = child internalising behaviour problems; s = slope factor.

*** *p* < 0.001.

** *p* < 0.01.

* *p* < 0.05.

#### 3.1.3 Covariation Between Mother–Child Closeness and Child Prosocial Behaviour

The results presented in Table 5 show that the trajectories of mother–child closeness and child prosocial behaviour positively covaried at Wave 1 and over time (intercept: *b* = 8.552, SE = 0.853, *p* < 0.001; slope: *b* = 0.878, SE = 0.143, *p* < 0.001), despite there being no significant average rates of change. Therefore, children with an increasingly close relationship with their mother displayed more prosocial behaviours over time (and vice versa). There was also significant variation across participants' trajectories of mother–child closeness and child prosocial behaviour at Wave 1 (closeness: *b* = 18.787, SE = 2.041, *p* < 0.001; prosocial: *b*= 6.205, SE = 0.621, *p* < 0.001) and over time (closeness: *b* = 1.742, SE = 0.822, *p* = 0.034; prosocial: *b* = 0.589, SE = 0.184, *p* = 0.001), suggesting that there may be some individual‐level change over time.

**TABLE 5:** Parameter estimates for unconditional parallel process growth modelling of trajectory of mother–child closeness and trajectory of child prosocial behaviour.

	Coefficient	Std. error	CI (95%)		*p*
			Lower	Upper	
Intercepts					
iCloseness	26.256	0.262	25.741	26.770	< 0.001***
iProsocial	3.835	0.146	3.548	4.121	< 0.001***
sCloseness	−0.464	2.580	−5.520	4.592	0.857
sProsocial	1.311	0.873	−0.399	3.022	0.133
Regressions					
sProsocial ~ iCloseness	−0.036	0.045	−0.125	0.053	0.425
sProsocial ~ iProsocial	−0.027	0.090	−0.203	0.149	0.762
sCloseness ~ iProsocial	−0.218	0.182	−0.575	0.138	0.230
sCloseness ~ iCloseness	0.060	0.123	−0.181	0.301	0.624
Variances					
iCloseness	18.787	2.041	14.787	22.786	< 0.001***
sCloseness	1.742	0.822	0.131	3.352	0.034*
iProsocial	6.205	0.621	4.988	7.423	< 0.001***
sProsocial	0.589	0.184	0.229	0.949	0.001***
Covariances					
iCloseness ~~ iProsocial	8.552	0.853	6.880	10.223	< 0.001***
sCloseness ~~ sProsocial	0.878	0.143	0.597	1.158	< 0.001***

Abbreviations: CI = confidence interval; Closeness = mother–child closeness; i = intercept factor; Prosocial = child prosocial behaviours; s = slope factor.

*** *p* < 0.001.

* *p* < 0.05.

### 3.2 Conditional Parallel Process Growth Models

**FIGURE 1:**
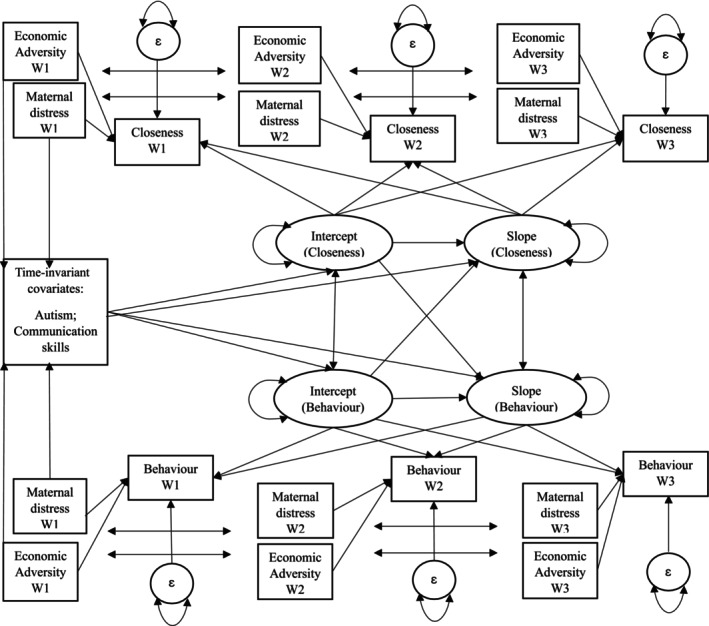
Path diagram for the conditional parallel process growth curve models examining the covarying relationship between the trajectories of mother–child closeness (Closeness) and child externalising behaviour problems, child internalising behaviour problems and child prosocial behaviour, respectively (Behaviour), as estimated using intercept and slope latent growth factors. Time‐varying covariates measured at each wave (W) include family economic adversity (Economic Adversity) and maternal psychological distress (Maternal Distress). Time‐invariant covariates include the diagnosis of autism (Autism) and the child's level of communication skills (Communication Skills).

Given the findings from the unconditional models, we continued to follow the pre‐registered plan to fit the conditional models to see if these covariates could partially explain the significant variation in each outcome over time. The findings from the model fit statistics indicated that all models displayed good fit to the data (see Tables S10–S13). Figure 1 displays the path diagram for the conditional parallel process growth models.

#### 3.2.1 Covariation Between Mother–Child Closeness and Child Externalising Behaviour Problems

As shown in Table 6, when adding the covariates into the model, we found a negative covarying relationship between mother–child closeness and child externalising behaviour problems at Wave 1 (*b* = −2.163, SE = 0.621, *p* < 0.001) but not over time, thus contrasting our finding from the unconditional model. The trajectories for both mother–child closeness and child externalising behaviour problems remained stable over time. Furthermore, although there was significant variation in mother–child closeness (*b* = 10.696, SE = 1.627, *p* < 0.001) and child externalising behaviour problems (*b* = 4.745, SE = 0.721, *p* < 0.001) at Wave 1, this variance did not continue over time in either outcome. In terms of the covariates, the child's level of communication skills and a diagnosis of autism were significantly associated with both mother–child closeness and child externalising behaviour problems at Wave 1. Family economic adversity was positively associated with child externalising behaviour problems only at Wave 1. Both the child's level of communication skills and maternal psychological distress were significantly associated with child externalising behaviour problems over time, whereas only maternal psychological distress was significantly associated with mother–child closeness at Waves 2 and 3.

**TABLE 6:** Parameter estimates for conditional parallel process growth modelling of trajectory of mother–child closeness and trajectory of child externalising behaviour problems.

	Coefficient	Std. error	CI (95%)		*p*
			Lower	Upper	
Intercepts					
iCloseness	22.876	0.956	21.003	24.749	< 0.001***
iExternal	7.557	0.619	6.344	8.770	< 0.001***
sCloseness	−0.892	2.567	−5.922	4.139	0.728
sExternal	−2.162	1.481	−5.064	0.740	0.144
Regressions					
sExternal ~ iCloseness	0.070	0.037	−0.002	0.143	0.058
sExternal ~ iExternal	0.126	0.101	−0.071	0.323	0.211
sCloseness ~ iExternal	0.056	0.082	−0.104	0.216	0.493
sCloseness ~ iCloseness	0.008	0.092	−0.172	0.188	0.931
iExternal ~ Autism	2.252	0.319	1.626	2.878	< 0.001***
iExternal ~ Communication	0.149	0.065	0.022	0.276	0.021*
sExternal ~ Autism	−0.163	0.246	−0.646	0.320	0.508
sExternal ~ Communication	−0.167	0.056	−0.277	−0.056	0.003**
iCloseness ~ Autism	−2.916	0.485	−3.868	−1.965	< 0.001***
iCloseness ~ Communication	0.949	0.099	0.755	1.142	< 0.001***
sCloseness ~ Autism	0.041	0.341	−0.627	0.710	0.904
sCloseness ~ Communication	0.013	0.108	−0.199	0.226	0.902
Closeness0 ~ Maternal Distress0	−0.048	0.038	−0.122	0.026	0.207
Closeness1 ~ Maternal Distress1	−0.154	0.037	−0.227	−0.081	< 0.001***
Closeness2 ~ Maternal Distress2	−0.157	0.040	−0.236	−0.079	< 0.001***
External0 ~ Maternal Distress0	0.082	0.023	0.037	0.128	< 0.001***
External1 ~ Maternal Distress1	0.129	0.025	0.080	0.178	< 0.001***
External2 ~ Maternal Distress2	0.154	0.025	0.106	0.203	< 0.001***
Closeness0 ~ FEA0	−0.216	0.111	−0.432	0.001	0.051
Closeness1 ~ FEA1	0.092	0.095	−0.093	0.278	0.328
Closeness2 ~ FEA2	0.113	0.115	−0.112	0.339	0.324
External0 ~ FEA0	0.139	0.068	0.005	0.273	0.042*
External1 ~ FEA1	0.047	0.063	−0.077	0.171	0.460
External2 ~ FEA2	−0.093	0.072	−0.235	0.049	0.198
Variances					
iCloseness	10.696	1.627	7.507	13.884	< 0.001***
sCloseness	0.943	0.800	−0.625	2.511	0.239
iExternal	4.745	0.721	3.332	6.157	< 0.001***
sExternal	0.030	0.430	−0.813	0.191	0.945
Covariances					
iCloseness ~~ iExternal	−2.163	0.621	−3.381	−0.945	< 0.001***
sCloseness ~~ sExternal	−0.127	0.172	−0.465	0.210	0.460

Abbreviations: CI = confidence interval; Closeness = mother–child closeness; External = child externalising behaviour problems; FEA = family economic adversity; i = intercept factor; s = slope factor.

*** *p* < 0.001.

** *p* < 0.01.

* *p* < 0.05.

#### 3.2.2 Covariation Between Mother–Child Closeness and Child Internalising Behaviour Problems

As presented in Table 7, the trajectories of mother–child closeness and child internalising behaviours remained stable and did not covary. However, there was a negative covarying relationship between mother–child closeness and child internalising behaviour problems at Wave 1 (*b* = −2.317, SE = 0.768, *p* = 0.003). Thus, families with a close mother–child relationship at Wave 1 displayed fewer child internalising behaviour problems (and vice versa). Child internalising behaviour problems at Wave 1 were associated with reduced child internalising behaviours over time (*b* = −0.163, SE = 0.052, *p* = 0.002). Upon adding the covariates into the model, the child's level of communication skills and a diagnosis of autism were associated with mother–child closeness and child internalising behaviour problems at Wave 1 only. Although maternal psychological distress and family economic adversity were positively associated with child internalising behaviour problems at Wave 1 and over time, only maternal psychological distress was negatively associated with mother–child closeness at Waves 2 and 3. Significant individual differences were present in both mother–child closeness and child internalising behaviour problems at Wave 1 (closeness: *b* = 11.381, SE = 1.700, *p* < 0.001; internalising: *b* = 8.704, SE = 1.129, *p* < 0.001) and in child internalising behaviour problems over time (*b* = 1.148, SE = 0.341, *p* = 0.001).

**TABLE 7:** Parameter estimates for conditional parallel process growth modelling of trajectory of mother–child closeness and trajectory of child internalising behaviour problems.

	Coefficient	Std. error	CI (95%)		*p*
			Lower	Upper	
Intercepts					
iCloseness	22.768	0.959	20.899	24.647	< 0.001***
iInternal	2.085	0.776	0.564	3.605	0.007**
sCloseness	0.626	1.898	−3.094	4.346	0.741
sInternal	0.372	0.867	−1.327	2.071	0.668
Regressions					
sInternal ~ iCloseness	0.028	0.032	−0.035	0.091	0.383
sInternal ~ iInternal	−0.163	0.052	−0.265	−0.061	0.002**
sCloseness ~ iInternal	0.017	0.049	−0.079	0.113	0.729
sCloseness ~ iCloseness	−0.040	0.078	−0.193	0.112	0.602
iInternal ~ Autism	3.325	0.395	2.552	4.098	< 0.001***
iInternal ~ Communication	0.350	0.080	0.193	0.507	< 0.001***
sInternal ~ Autism	0.465	0.241	−0.008	0.938	0.054
sInternal ~ Communication	0.035	0.055	−0.072	0.143	0.516
iCloseness ~ Autism	−2.911	0.486	−3.864	−1.958	< 0.001***
iCloseness ~ Communication	0.945	0.099	0.751	1.139	< 0.001***
sCloseness ~ Autism	−0.031	0.326	−0.670	0.609	0.925
sCloseness ~ Communication	0.062	0.097	−0.127	0.252	0.520
Closeness0 ~ Maternal Distress0	−0.043	0.038	−0.116	0.031	0.258
Closeness1 ~ Maternal Distress1	−0.150	0.038	−0.223	−0.076	< 0.001***
Closeness2 ~ Maternal Distress2	−0.150	0.040	−0.228	−0.072	< 0.001***
Internal0 ~ Maternal Distress0	0.092	0.030	0.033	0.150	0.002**
Internal1 ~ Maternal Distress1	0.145	0.030	0.087	0.204	< 0.001***
Internal2 ~ Maternal Distress2	0.102	0.030	0.042	0.161	0.001***
Closeness0 ~ FEA0	−0.200	0.110	−0.417	0.016	0.070
Closeness1 ~ FEA1	0.102	0.095	−0.085	0.290	0.284
Closeness2 ~ FEA2	0.111	0.115	0.337	0.111	0.336
Internal0 ~ FEA0	0.370	0.088	0.197	0.542	< 0.001***
Internal1 ~ FEA1	0.233	0.075	0.085	0.381	0.002**
Internal2 ~ FEA2	0.217	0.087	0.046	0.388	0.013*
Variances					
iCloseness	11.381	1.700	8.049	14.714	< 0.001***
sCloseness	1.395	0.727	−0.031	2.820	0.055
iInternal	8.704	1.129	6.492	10.916	< 0.001***
sInternal	1.148	0.341	0.480	1.815	0.001***
Covariances					
iCloseness ~~ iInternal	−2.317	0.768	−3.822	−0.813	0.003**
sCloseness ~~ sInternal	0.235	0.174	−0.106	0.576	0.177

Abbreviations: CI = confidence interval; Closeness = mother–child closeness; FEA = family economic adversity; i = intercept factor; Internal = child internalising behaviour problems; s = slope factor.

*** *p* < 0.001.

** *p* < 0.01.

* *p* < 0.05.

#### 3.2.3 Covariation Between Mother–Child Closeness and Child Prosocial Behaviour

As displayed in Table 8, the trajectories of mother–child closeness and child prosocial behaviour continued to positively covary at Wave 1 and over time (intercept: *b* = 4.321, SE = 0.552, *p* < 0.001; slope: *b* = 0.754, SE = 0.152, *p* < 0.001), despite there being no significant average rates of change over time. When adding the covariates into the model, we found that the child's level of communication skills, a diagnosis of autism and family economic adversity were significantly associated with mother–child closeness at Wave 1. All covariates except for family economic adversity were significantly associated with child prosocial behaviour at Wave 1 and over time; family economic adversity was associated with child prosocial behaviour at Waves 1 and 2 only. Maternal psychological distress was associated with mother–child closeness at Waves 2 and 3. In contrast to the unconditional model, there was significant variation in mother–child closeness at Wave 1 (*b* = 11.478, SE = 1.623, *p* < 0.001) but not over time. Therefore, this finding suggests that the added covariates accounted for these individual differences in mother–child closeness over time. However, as there was significant variation in child prosocial behaviour at Wave 1 and over time (intercept: *b* = 3.494, SE = 0.449, *p* < 0.001; slope: *b* = 0.414, SE = 0.179, *p* = 0.020), individual differences in the trajectory growth of child prosocial behaviour may be more important than overall model growth.

**TABLE 8:** Parameter estimates for conditional parallel process growth modelling of trajectory of mother–child closeness and trajectory of child prosocial behaviour.

	Coefficient	Std. error	CI (95%)		*p*
			Lower	Upper	
Intercepts					
iCloseness	22.884	0.955	21.012	24.755	< 0.001***
iProsocial	1.652	0.507	0.660	2.645	0.001***
sCloseness	−1.598	3.029	−7.534	4.338	0.598
sProsocial	1.638	1.027	−0.375	3.650	0.111
Regressions					
sProsocial ~ iCloseness	−0.081	0.050	−0.178	0.016	0.103
sProsocial ~ iProsocial	−0.009	0.109	−0.222	0.205	0.936
sCloseness ~ iProsocial	−0.333	0.200	−0.726	0.059	0.096
sCloseness ~ iCloseness	0.080	0.142	−0.198	0.359	0.571
iProsocial ~ Autism	−1.337	0.260	−1.846	−0.828	< 0.001***
iProsocial ~ Communication	0.585	0.053	0.482	0.688	< 0.001***
sProsocial ~ Autism	−0.411	0.145	−0.695	−0.128	0.004**
sProsocial ~ Communication	0.097	0.040	0.019	0.175	0.014*
iCloseness ~ Autism	−2.878	0.487	−3.833	−1.924	< 0.001***
iCloseness ~ Communication	0.941	0.099	0.747	1.135	< 0.001***
sCloseness ~ Autism	−0.069	0.331	−0.718	0.579	0.834
sCloseness ~ Communication	0.149	0.077	−0.001	0.299	0.052
Closeness0 ~ Maternal Distress0	−0.039	0.038	−0.113	0.035	0.299
Closeness1 ~ Maternal Distress1	−0.152	0.037	−0.225	−0.079	< 0.001***
Closeness2 ~ Maternal Distress2	−0.155	0.040	−0.233	−0.077	< 0.001***
Prosocial0 ~ Maternal Distress0	−0.046	0.020	−0.085	−0.007	0.019*
Prosocial1 ~ Maternal Distress1	−0.046	0.019	−0.083	−0.009	0.016*
Prosocial2 ~ Maternal Distress2	−0.063	0.020	−0.101	−0.024	0.001***
Closeness0 ~ FEA0	−0.230	0.109	−0.444	−0.016	0.036*
Closeness1 ~ FEA1	0.095	0.094	−0.090	0.279	0.314
Closeness2 ~ FEA2	0.119	0.114	−0.105	0.343	0.299
Prosocial0 ~ FEA0	−0.148	0.057	−0.261	−0.036	0.010**
Prosocial1 ~ FEA1	−0.015	0.048	−0.108	0.079	0.016*
Prosocial2 ~ FEA2	0.093	0.056	−0.017	0.203	0.098
Variances					
iCloseness	11.478	1.623	8.297	14.659	< 0.001***
sCloseness	1.132	0.832	−0.499	2.763	0.174
iProsocial	3.494	0.449	2.613	4.374	< 0.001***
sProsocial	0.414	0.179	0.064	0.764	0.020*
Covariances					
iCloseness ~~ iProsocial	4.321	0.552	3.238	5.403	< 0.001***
sCloseness ~~ sProsocial	0.754	0.152	0.456	1.053	< 0.001***

Abbreviations: CI = confidence interval; Closeness = mother–child closeness; FEA = family economic adversity; i = intercept factor; Prosocial = child prosocial behaviour; s = slope factor.

*** *p* < 0.001.

** *p* < 0.01.

* *p* < 0.05.

## 4 Discussion

This study aimed to examine whether the trajectory of mother–child closeness covaried with the trajectory of child behaviour (externalising behaviour problems, internalising behaviour problems and prosocial behaviour). When controlling for the effects of time‐varying and time‐invariant covariates, we found that the trajectory of mother–child closeness and child prosocial behaviour positively covaried over time. Although the trajectory of mother–child closeness did not covary with the trajectory of child internalising or externalising behaviour problems, we did find a covarying relationship was present for both outcomes at Wave 1.

The positive covariation between the trajectories of mother–child closeness and child prosocial behaviour suggests that children with intellectual disabilities who share an increasingly close relationship with their mother are also likely to display increases in prosocial behaviour over time (and vice versa). This finding may reflect reciprocal processes between the mother and child, whereby a close relationship with the mother facilitates the modelling of prosocial behaviour for their child, which, in turn, may enhance mother–child interactions and thus a closer relationship (Bronfenbrenner and Ceci 1994). Therefore, this aligns with the theoretical perspective that a close parent–child relationship is important for children's socioemotional outcomes (Bowlby 1977; Bronfenbrenner and Ceci 1994). Although the current study is novel in its longitudinal and reciprocal focus, the association between these variables extends previous findings reported by Williams et al. (2025), who found a positive association between parent–child closeness and child prosocial behaviour when examining time‐specific directional associations. Despite accounting for covariates in the model, we continued to find significant heterogeneity in child prosocial behaviour over time. Thus, individual differences in child prosocial behaviour may be more important for understanding this covarying relationship than overall model growth. Future research should therefore consider examining distinct subgroup trajectories of prosocial behaviour in families of children with intellectual disabilities, whilst considering the influence of factors such as maternal psychological distress, the child's level of communication skills and a diagnosis of autism, which were identified in this study as significantly contributing to the development of this behaviour over time.

Our findings indicated that the trajectory of mother–child closeness did not covary with the trajectory of child internalising or externalising behaviour problems when controlling for covariate factors. However, we found a negative covariation between mother–child closeness and child externalising and internalising behaviour problems at Wave 1, suggesting that children with increased levels of mother–child closeness at Wave 1 displayed fewer child internalising and externalising behaviour problems (and vice versa). The presence of a concurrent negative covariation at Wave 1 is consistent with previous research examining the unidirectional association between closeness and child behavioural problems in families of children with intellectual disabilities (Totsika et al. 2014). Conversely, the absence of a covariation between these factors over time might instead suggest that the relationship between these factors is indeed a time‐specific directional association, in which early mother–child closeness is predictive of later child behavioural outcomes (Schuiringa et al. 2015; Totsika et al. 2014). As the current study did not control for age effects, future research should draw upon data from a large population‐based birth cohort study to consider whether the child's stage of development influences this covariation over time.

A recurring finding across all parallel process models was that the trajectories of mother–child closeness and child behaviour (externalising behaviour problems, internalising behaviour problems and prosocial behaviour) remained relatively stable. Thus, individual trajectory growth may be more important than overall model growth. The stability in the trajectory of mother–child closeness is consistent with findings by Fielding‐Gebhardt et al. (2020), who examined this trajectory growth in families of children with Fragile X syndrome. In contrast, previous findings in the typically developing literature have suggested that mother–child closeness tends to decline over time during childhood and into adolescence (Nomaguchi and Allen 2023; Shanahan et al. 2007; Xie et al. 2021; Yan et al. 2019). This consistent finding with Fielding‐Gebhardt et al. may suggest that mother–child closeness develops differently in families of children with intellectual disabilities, potentially due to the mother's ongoing caregiving role, whereas typically developing children increasingly gain independence and form close relationships (e.g., peer and romantic relations) outside of the family, which may contribute to a less close mother–child relationship over time. Average rates of change in mother–child closeness may therefore occur over a longer period in families of children with intellectual disabilities than in families of non‐disabled children.

In terms of child behaviour, our finding that child externalising behaviour problems remained stable over time contrasts with previous research reporting improvements in this behaviour over time (Bailey et al. 2019; De Ruiter et al. 2007; Einfeld et al. 2006). This difference may be because these previous studies drew upon data from birth population cohort studies and controlled for age and developmental effects. Furthermore, these studies analysed data collected at four time points, which enabled the identification of non‐linear trajectory patterns. Consistent with Bailey et al. (2019) and De Ruiter et al. (2007), child internalising behaviour problems in our study also remained stable over time. One possibility for this finding is that, as data were parent‐reported, internalising behaviour problems may be more difficult to identify in this group of children than externalising behaviour problems. Moreover, unlike Bailey et al., who found improvements in child prosocial behaviour over time, child prosocial behaviour remained relatively stable over time in the current study, which may similarly reflect our inability to control for age effects and examine the development of this behaviour across more than three waves of data.

The inclusion of child, maternal and environmental covariates was important for examining the covarying relationship between mother–child closeness and child behavioural and emotional outcomes over time (Bronfenbrenner and Ceci 1994). In the conditional models, the covariates collectively accounted for the covariation in mother–child closeness and child externalising behaviour problems over time, indicating that there may instead be an indirect covarying relationship explained by these underlying factors. Further, the findings suggested that child characteristics (diagnosis of autism and the child's level of communication skills) appeared to be more important for the development of initial levels of mother–child closeness, whereas maternal psychological distress was more influential in shaping levels of mother–child closeness over time. The association between the proposed covariates and closeness supports previous findings in the intellectual disability literature (Chadwick et al. 2008; Howe 2006; Sterkenberg et al. 2022; Teague et al. 2020; Totsika et al. 2020; Zabidi et al. 2023). Regarding child behaviour, child, maternal and environmental characteristics collectively explained children's behavioural and emotional outcomes at Wave 1 in all models. Only maternal psychological distress was significantly associated with child behaviour over time in all three models, supporting previous findings that maternal well‐being is particularly important for shaping the development of children's behavioural and emotional outcomes (Hastings et al. 2006; Neece et al. 2012).

### 4.1 Strengths and Limitations

The current study was novel in its attempt to examine the covarying relationship between the trajectories of mother–child closeness and children's behavioural and emotional outcomes in families of children with intellectual disabilities. As previous research has typically examined this relationship in the context of the influence of mother–child closeness on later child outcomes, this study offers a starting point for understanding the reciprocal relationship between these factors over time in these families. Although this study drew upon data collected as part of a large UK longitudinal cohort study involving families of children with intellectual disabilities, data were only available at three time points, and thus limited our findings to linear trajectories only. With additional time points, research could examine for different trajectory patterns (e.g., cubic and quadratic) and whether average rates of change in mother–child closeness do indeed occur over a longer period of time. Furthermore, most participants in the current study identified as White British and were educated to degree level, which may not be representative of all families with children with intellectual disabilities in the UK and thus limits the generalisability of these findings. A further limitation of the current study was that data were gathered using parent‐reported measures. To enhance the reliability of these findings, it would have been useful to incorporate other methods such as observations of mother–child interactions, which have been used in previous studies examining facets of the parent–child relationship (e.g., Fenning et al. 2007; Fenning et al. 2014; Norona and Baker 2017; Rodas et al. 2016). Lastly, using the short‐form version of the CPRS may have been a limitation in this study, particularly for detecting any longitudinal change in mother–child closeness.

### 4.2 Practical Implications

The finding that there is a covarying relationship between mother–child closeness and child prosocial behaviour suggests that interventions aiming to improve the quality of mother–child relationship and/or child prosocial behaviour could benefit from considering the reciprocal relationship between these factors. Such interventions may be particularly important for mothers experiencing greater levels of psychological distress, children with lower communication skills and children who are autistic in addition to an intellectual disability. Nevertheless, these findings highlight the need for further research to better understand this covarying relationship. Future research could draw upon birth population cohort data with additional time points to control for age effects and examine underlying individual differences within these families.

## Funding

This study was supported by the Economic and Social Research Council Midlands Graduate School Doctoral Training Partnership as part of a funded PhD studentship (ES/P00711/1). The 1000 Families Study was funded by the following organisations: the University of Warwick, the Economic and Social Research Council Warwick Doctoral Training Centre and Cerebra UK.

## Ethics Statement

This study received ethics approval from the Humanities and Social Sciences Research Ethics Committee at the University of Warwick (HSSREC.147/23‐24).

## Conflicts of Interest

The authors declare no conflicts of interest.

## Supporting information


**Table S1:** Model Fit Statistics: Univariate Growth Curve Models.
**Table S2:** Growth Parameter Estimates for Mother–Child Closeness Univariate Growth Curve Model.
**Table S3:** Growth Parameter Estimates for Child Externalising Behaviour Problems Univariate Growth Curve Model.
**Table S4:** Growth Parameter Estimates for Child Internalising Behaviour Problems Univariate Growth Curve Model.
**Table S5:** Growth Parameter Estimates for Child Prosocial Behaviour Univariate Growth Curve Model.
**Table S6:** Model Fit Indices for Unconditional Parallel Process Growth Models.
**Table S7:** Parameter Estimates for Unconditional Parallel Process Growth Modelling of Trajectory of Mother–Child Closeness and Trajectory of Child Externalising Behaviour Problems.
**Table S8:** Parameter Estimates for Unconditional Parallel Process Growth Modelling of Trajectory of Mother–Child Closeness and Trajectory of Child Internalising Behaviour Problems.
**Table S9:** Parameter Estimates for Unconditional Parallel Process Growth Modelling of Trajectory of Mother–Child Closeness and Trajectory of Child Prosocial Behaviour.
**Table S10:** Model Fit Indices for Conditional Parallel Process Growth Models.
**Table S11:** Parameter Estimates for Conditional Parallel Process Growth Modelling of Trajectory of Mother–Child Closeness and Trajectory of Child Externalising Behaviour Problems.
**Table S12:** Parameter Estimates for Conditional Parallel Process Growth Modelling of Trajectory of Mother–Child Closeness and Trajectory of Child Internalising Behaviour Problems.
**Table S13:** Parameter Estimates for Conditional Parallel Process Growth Modelling of Trajectory of Mother–Child Closeness and Child Prosocial Behaviour.

## Data Availability

Due to ethical approval requirements, data are not available for sharing. Researchers interested in collaboration should contact Professor Richard Hastings.
