# Supplementary material for: Trajectories of Mother–Child Closeness and Child Behavioural and Emotional Outcomes in Families of Children With Intellectual Disabilities
Source: J Intellect Disabil Res. 2026 May 8;70(7):709–24. doi: 10.1111/jir.70110 (PMC13238317; doi:10.1111/jir.70110)
Supplement: Supplementary file 1 — Table S1: Model Fit Statistics: Univariate Growth Curve Models. Table S2: Growth Parameter Estimates for Mother–Child Closeness Univariate Growth Curve Model. Table S3: Growth Parameter Estimates for Child Externalising Behaviour Problems Univariate Growth Curve Model. Table S4: Growth Parameter Estimates for Child Internalising Behaviour Problems Univariate Growth Curve Model. Table S5: Growth Parameter Estimates for Child Prosocial Behaviour Univariate Growth Curve Model. Table S6: Model Fit Indices for Unconditional Parallel Process Growth Models. Table S7: Parameter Estimates for Unconditional Parallel Process Growth Modelling of Trajectory of Mother–Child Closeness and Trajectory of Child Externalising Behaviour Problems. Table S8: Parameter Estimates for Unconditional Parallel Process Growth Modelling of Trajectory of Mother–Child Closeness and Trajectory of Child Internalising Behaviour Problems. Table S9: Parameter Estimates for Unconditional Parallel Process Growth Modelling of Trajectory of Mother–Child Closeness and Trajectory of Child Prosocial Behaviour. Table S10: Model Fit Indices for Conditional Parallel Process Growth Models. Table S11: Parameter Estimates for Conditional Parallel Process Growth Modelling of Trajectory of Mother–Child Closeness and Trajectory of Child Externalising Behaviour Problems. Table S12: Parameter Estimates for Conditional Parallel Process Growth Modelling of Trajectory of Mother–Child Closeness and Trajectory of Child Internalising Behaviour Problems. Table S13: Parameter Estimates for Conditional Parallel Process Growth Modelling of Trajectory of Mother–Child Closeness and Child Prosocial Behaviour. [file JIR-70-709-s001.pdf]

**Supplementary Material**

**Table S1**

*Model Fit Statistics: Univariate Growth Curve Models*

| **Model** | **$X^2$ (df)** | ***p*** | **CFI** | **TLI** | **SRMR** | **RMSEA** | **RMSEA 95% CI** | | **RMSEA** |
|---|---|---|---|---|---|---|---|---|---|
| | | | | | | | **Lower** | **Upper** | ***p*** |
| Mother-Child Closeness | 5.567 (1) | 0.018* | 0.991 | 0.973 | 0.022 | 0.118 | 0.039 | 0.221 | .073 |
| Child Externalising Behaviour Problems | 2.854 (1) | 0.091 | 0.996 | 0.988 | 0.017 | 0.075 | 0.000 | 0.184 | .221 |
| Child Internalising Behaviour Problems | 2.125 (1) | 0.145 | 0.998 | 0.993 | 0.014 | 0.059 | 0.000 | 0.172 | .299 |
| Child Prosocial Behaviour | 3.410(1) | 0.065 | 0.996 | 0.988 | 0.016 | 0.086 | 0.000 | 0.193 | .176 |

*Note.* *$p$= <0.05; $X^2$ = chi squared, df = degrees of freedom; CFI = Comparative Fit Index; TLI = Tucker Lewis Index; RMSEA = Root Mean Square Error of Approximation; CI = Confidence Intervals; SRMR = Standardised Root Mean Square Residual.

**Table S2**

*Growth Parameter Estimates for Mother-Child Closeness Univariate Growth Curve Model*

| | **Coefficient** | **Std. Error** | ***p*** |
|---|---|---|---|
| **Covariances** | | | |
| iCloseness ~~ sCloseness | -1.062 | 1.048 | .311 |
| **Intercepts** | | | |
| Closeness0 | 0.000 | | |
| Closeness1 | 0.000 | | |
| Closeness2 | 0.000 | | |
| iCloseness | 26.329 | 0.269 | <.001*** |
| sCloseness | 0.271 | 0.122 | .027* |
| **Variances** | | | |
| Closeness0 | 4.842 | 1.744 | .006** |
| Closeness1 | 7.665 | 1.011 | <.001*** |
| Closeness2 | 4.588 | 1.930 | .017* |
| iCloseness | 19.393 | 2.280 | <.001*** |
| sCloseness | 2.529 | 0.918 | .006** |

*Note.* ***$p$ = <0.001, **$p$ = <0.01, *$p$= <0.05; i = intercept factor; s = slope factor

**Table S3**

*Growth Parameter Estimates for Child Externalising Behaviour Problems Univariate Growth Curve Model*

| | **Coefficient** | **Std. Error** | ***p*** |
|---|---|---|---|
| **Covariances** | | | |
| iExternal ~~ sExternal | -0.087 | 0.444 | .844 |
| **Intercepts** | | | |
| External0 | 0.000 | | |
| External1 | 0.000 | | |
| External2 | 0.000 | | |
| iExternal | 11.570 | 0.168 | <.001*** |
| sExternal | -0.430 | 0.074 | <.001*** |
| **Variances** | | | |
| External0 | 2.206 | 0.757 | .004** |
| External 1 | 3.627 | 0.455 | <.001*** |
| External 2 | 2.612 | 0.848 | .002** |
| i External | 7.232 | 0.929 | <.001*** |
| s External | 0.579 | 0.392 | .140 |

*Note.* ****p* = <0.001, ***p* = <0.01; i = intercept factor; s = slope factor

**Table S4**

*Growth Parameter Estimates for Child Internalising Behaviour Problems Univariate Growth Curve Model*

| | **Coefficient** | **Std. Error** | ***p*** |
|---|---|---|---|
| **Covariances** | | | |
| iInternal ~~ sInternal | -1.822 | 0.692 | .008** |
| **Intercepts** | | | |
| Internal0 | 0.000 | | |
| Internal1 | 0.000 | | |
| Internal2 | 0.000 | | |
| iInternal | 9.397 | 0.222 | <.001*** |
| sInternal | 0.302 | 0.094 | .001*** |
| **Variances** | | | |
| Internal0 | 2.371 | 1.105 | .032* |
| Internal1 | 4.599 | 0.606 | <.001*** |
| Internal2 | 3.261 | 1.098 | .003** |
| iInternal | 14.026 | 1.546 | <.001*** |
| sInternal | 1.494 | 0.549 | .007** |

*Note.* ****p* = <0.001, ***p* = <0.01, **p*= <0.05; i = intercept factor; s = slope factor

**Table S5**

*Growth Parameter Estimates for Child Prosocial Behaviour Univariate Growth Curve Model*

| | **Coefficient** | **Std. Error** | ***p*** |
|---|---|---|---|
| **Covariances** | | | |
| iProsocial ~~ sProsocial | -0.512 | 0.290 | .078 |
| **Intercepts** | | | |
| Prosocial0 | 0.000 | | |
| Prosocial1 | 0.000 | | |
| Prosocial2 | 0.000 | | |
| iProsocial | 3.842 | 0.150 | <.001*** |
| sProsocial | 0.266 | 0.062 | <.001*** |
| **Variances** | | | |
| Prosocial0 | 1.254 | 0.488 | .010** |
| Prosocial1 | 1.986 | 0.271 | <.001*** |
| Prosocial2 | 0.901 | 0.501 | .072 |
| iProsocial | 6.321 | 0.687 | <.001*** |
| sProsocial | 0.723 | 0.247 | .003** |

*Note.* ***$p$ = <0.001, **$p$ = <0.01; i = intercept factor; s = slope factor

**Table S6**

*Model fit indices for Unconditional Parallel Process Growth Models.*

| Model | $\chi^2$ (df) | *p* | CFI | TLI | SRMR | RMSEA | RMSEA 95% CI | | RMSEA |
|---|---|---|---|---|---|---|---|---|---|
| | | | | | | | Lower | Upper | *p* |
| Model 1 | 14.943(6) | 0.021* | 0.991 | 0.979 | 0.027 | 0.065 | 0.024 | 0.107 | .233 |
| Model 2 | 14.929 (7) | 0.037* | 0.993 | 0.984 | 0.020 | 0.057 | 0.013 | 0.097 | .341 |
| Model 3 | 8.714(6) | 0.190 | 0.998 | 0.995 | 0.019 | 0.036 | 0.000 | 0.084 | .067 |

*Note.* *$p$= <0.05; $\chi^2$ = Chi Squared, df = degrees of freedom; CFI = Comparative Fit Index; TLI = Tucker Lewis Index; RMSEA = Root Mean Square Error of Approximation; CI = Confidence Intervals; SRMR = Standardised Root Mean Square Residual; Model 1 = Mother-Child Closeness and Child Externalising Behaviour Problems; Model 2 = Mother-Child Closeness and Child Internalising Behaviour Problems; Model 3 = Mother-Child Closeness and Child Prosocial Behaviour

**Table S7**

*Parameter Estimates for Unconditional Parallel Process Growth Modelling of Trajectory of Mother-Child Closeness and Trajectory of Child Externalising Behaviour Problems*

| | Coefficient | Std. Error | CI (95%) Lower | CI (95%) Upper | *p* |
|---|---|---|---|---|---|
| **Intercepts** | | | | | |
| iCloseness | 26.302 | 0.263 | 25.786 | 26.819 | <.001*** |
| iExternal | 11.572 | 0.164 | 11.250 | 11.894 | <.001*** |
| sCloseness | -0.369 | 2.120 | -4.524 | 3.785 | .862 |
| sExternal | -0.780 | 1.370 | -3.465 | 1.905 | .569 |
| **Regressions** | | | | | |
| sExternal ~ iCloseness | 0.007 | 0.025 | -0.042 | 0.057 | .773 |
| sExternal ~ iExternal | 0.014 | 0.073 | -0.129 | 0.158 | .845 |
| sCloseness ~ iExternal | 0.082 | 0.063 | -0.042 | 0.206 | .196 |
| sCloseness ~ iCloseness | -0.011 | 0.061 | -0.131 | 0.109 | .852 |
| **Variances** | | | | | |
| iCloseness | 18.365 | 2.131 | 14.189 | 22.541 | <.001*** |
| sCloseness | 1.737 | 0.877 | 0.019 | 3.455 | .048* |
| iExternal | 6.918 | 0.894 | 5.166 | 8.670 | <.001*** |
| sExternal | 0.319 | 0.397 | -0.460 | 1.098 | .422 |
| Closeness0 | 6.105 | 1.703 | 2.766 | 9.443 | <.001*** |
| Closeness1 | 7.189 | 0.942 | 5.343 | 9.035 | <.001*** |
| Closeness2 | 6.130 | 1.861 | 2.483 | 9.777 | .001*** |
| External0 | 2.633 | 0.751 | 1.161 | 4.105 | <.001*** |
| External1 | 3.566 | 0.431 | 2.722 | 4.411 | <.001*** |

| | | | | | |
|---|---|---|---|---|---|
| External2 | 3.303 | 0.823 | 1.690 | 4.916 | <.001*** |
| **Covariances** | | | | | |
| iCloseness ~~ iExternal | -3.779 | 0.825 | -5.397 | -2.162 | <.001*** |
| sCloseness ~~ sExternal | -0.401 | 0.165 | -0.724 | -0.077 | .015* |

*Note.* ****p* = <0.001, **p*= <0.05; s=slope factor; i=intercept factor; External=Child Externalising Behaviour Problems; Closeness = Mother-Child Closeness; CI = confidence intervals

**Table S8**

*Parameter Estimates for Unconditional Parallel Process Growth Modelling of Trajectory of Mother-Child Closeness and Trajectory of Child Internalising Behaviour Problems*

| | Coefficient | Std. Error | CI (95%) | | $p$ |
|---|---|---|---|---|---|
| | | | Lower | Upper | |
| **Intercepts** | | | | | |
| iCloseness | 26.249 | 0.263 | 25.733 | 26.765 | <.001*** |
| iInternal | 9.491 | 0.217 | 9.066 | 9.917 | <.001*** |
| sCloseness | 1.621 | 1.561 | -1.439 | 4.681 | .299 |
| sInternal | 0.899 | 0.890 | -0.846 | 2.643 | .313 |
| **Regressions** | | | | | |
| sInternal ~ iCloseness | 0.014 | 0.023 | -0.032 | 0.060 | .540 |
| sInternal ~ iInternal | -0.104 | 0.045 | -0.191 | -0.017 | .020* |
| sCloseness ~ iInternal | 0.021 | 0.038 | -0.054 | 0.095 | .583 |
| sCloseness ~ iCloseness | -0.059 | 0.052 | -0.160 | 0.043 | .260 |
| **Variances** | | | | | |
| iCloseness | 19.465 | 2.252 | 15.051 | 23.880 | <.001*** |
| sCloseness | 2.410 | 0.812 | 0.817 | 4.002 | .003** |
| iInternal | 13.586 | 1.461 | 10.722 | 16.449 | <.001*** |
| sInternal | 1.055 | 0.444 | 0.185 | 1.924 | .017* |
| Closeness0 | 4.650 | 1.716 | 1.287 | 8.013 | .007** |
| Closeness1 | 7.840 | 0.990 | 5.901 | 9.780 | <.001*** |
| Closeness2 | 4.784 | 1.886 | 1.087 | 8.480 | .011* |
| Internal0 | 2.958 | 1.082 | 0.838 | 5.079 | .006* |
| Internal1 | 4.304 | 0.575 | 3.178 | 5.431 | <.001*** |

| | | | | | |
|---|---|---|---|---|---|
| Internal2 | 3.717 | 1.085 | 1.590 | 5.844 | .001*** |
| **Covariances** | | | | | |
| iCloseness ~~ iInternal | -4.115 | 1.078 | -6.228 | -2.001 | <.001*** |
| sCloseness ~~ sInternal | 0.125 | 0.182 | -0.231 | 0.482 | .490 |

*Note.* ***p = <0.001, **p=<0.01 *p= <0.05; s=slope factor; i=intercept factor; Internal=Child Internalising Behaviour Problems; Closeness = Mother-Child Closeness; CI = confidence intervals

**Table S9**

*Parameter Estimates for Unconditional Parallel Process Growth Modelling of Trajectory of Mother-Child Closeness and Trajectory of Child Prosocial Behaviour*

| | Coefficient | Std. Error | CI (95%) Lower | CI (95%) Upper | *p* |
|---|---|---|---|---|---|
| **Intercepts** | | | | | |
| iCloseness | 26.256 | 0.262 | 25.741 | 26.770 | <.001*** |
| iProsocial | 3.835 | 0.146 | 3.548 | 4.121 | <.001*** |
| sCloseness | -0.464 | 2.580 | -5.520 | 4.592 | .857 |
| sProsocial | 1.311 | 0.873 | -0.399 | 3.022 | .133 |
| **Regressions** | | | | | |
| sProsocial ~ iCloseness | -0.036 | 0.045 | -0.125 | 0.053 | .425 |
| sProsocial ~ iProsocial | -.027 | 0.090 | -0.203 | 0.149 | .762 |
| sCloseness ~ iProsocial | -.218 | 0.182 | -0.575 | 0.138 | .230 |
| sCloseness ~ iCloseness | .060 | 0.123 | -0.181 | 0.301 | .624 |
| **Variances** | | | | | |
| iCloseness | 18.787 | 2.041 | 14.787 | 22.786 | <.001*** |
| sCloseness | 1.742 | 0.822 | 0.131 | 3.352 | .034* |
| iProsocial | 6.205 | 0.621 | 4.988 | 7.423 | <.001*** |
| sProsocial | 0.589 | 0.184 | 0.229 | 0.949 | .001*** |
| Closeness0 | 5.697 | 1.367 | 3.017 | 8.377 | <.001*** |
| Closeness1 | 7.255 | 0.814 | 5.661 | 8.850 | <.001*** |
| Closeness2 | 6.153 | 1.531 | 3.152 | 9.155 | <.001*** |
| Prosocial0 | 1.350 | 0.371 | 0.623 | 2.077 | <.001*** |
| Prosocial1 | 1.924 | 0.220 | 1.493 | 2.354 | <.001*** |

| | | | | | |
|---|---|---|---|---|---|
| Prosocial2 | 1.113 | 0.387 | 0.355 | 1.872 | .004** |
| **Covariances** | | | | | |
| iCloseness ~~ iProsocial | 8.552 | 0.853 | 6.880 | 10.223 | <.001*** |
| sCloseness ~~ sProsocial | 0.878 | 0.143 | 0.597 | 1.158 | <.001*** |

*Note.* ****p* = <0.001, **p=<0.01 **p*= <0.05; s=slope factor; i=intercept factor; Prosocial=Child Prosocial Behaviour; Closeness = Mother-Child Closeness; CI = confidence intervals

**Table S10**

*Model fit indices for Conditional Parallel Process Growth Models.*

| Model | $X^2$ (df) | *p* | CFI | TLI | SRMR | RMSEA | RMSEA 90% CI | | RMSEA |
|---|---|---|---|---|---|---|---|---|---|
| | | | | | | | Lower | Upper | *p* |
| Model 1 | 41.082(34) | .188 | 0.995 | 0.990 | 0.029 | 0.025 | 0.000 | 0.050 | .953 |
| Model 2 | 51.380(34) | .028* | 0.987 | 0.976 | 0.035 | 0.040 | 0.013 | 0.061 | .775 |
| Model 3 | 41.597(34) | .174 | 0.995 | 0.991 | 0.018 | 0.026 | 0.000 | 0.050 | .948 |

*Note.* **p*= <0.05; $X^2$ = Chi Squared, df = degrees of freedom; CFI = Comparative Fit Index; TLI = Tucker Lewis Index; RMSEA = Root Mean Square Error of Approximation; CI = Confidence Intervals; SRMR = Standardised Root Mean Square Residual; Model 1 = Mother-Child Closeness and Child Externalising Behaviour Problems; Model 2 = Mother-Child Closeness and Child Internalising Behaviour Problems; Model 3 = Mother-Child Closeness and Child Prosocial Behaviour

**Table S11**

*Parameter Estimates for Conditional Parallel Process Growth Modelling of Trajectory of Mother-Child Closeness and Trajectory of Child Externalising Behaviour Problems*

| | Coefficient | Std. Error | CI (95%) Lower | CI (95%) Upper | *p* |
|---|---|---|---|---|---|
| **Intercepts** | | | | | |
| Closeness0 | 0.000 | | | | |
| Closeness1 | 0.000 | | | | |
| Closeness2 | 0.000 | | | | |
| External0 | 0.000 | | | | |
| External1 | 0.000 | | | | |
| External2 | 0.000 | | | | |
| iCloseness | 22.876 | 0.956 | 21.003 | 24.749 | <.001*** |
| iExternal | 7.557 | 0.619 | 6.344 | 8.770 | <.001*** |
| sCloseness | -0.892 | 2.567 | -5.922 | 4.139 | .728 |
| sExternal | -2.162 | 1.481 | -5.064 | 0.740 | .144 |
| **Regressions** | | | | | |
| sExternal ~ iCloseness | 0.070 | 0.037 | -0.002 | 0.143 | .058 |
| sExternal ~ iExternal | 0.126 | 0.101 | -0.071 | 0.323 | .211 |
| sCloseness ~ iExternal | 0.056 | 0.082 | -0.104 | 0.216 | .493 |
| sCloseness ~ iCloseness | 0.008 | 0.092 | -0.172 | 0.188 | .931 |
| iExternal ~ Autism | 2.252 | 0.319 | 1.626 | 2.878 | <.001*** |
| iExternal ~ Communication | 0.149 | 0.065 | 0.022 | 0.276 | .021* |
| sExternal ~ Autism | -0.163 | 0.246 | -0.646 | 0.320 | .508 |

| | | | | | |
|---|---|---|---|---|---|
| sExternal ~ Communication | -0.167 | 0.056 | -0.277 | -0.056 | .003** |
| iCloseness ~ Autism | -2.916 | 0.485 | -3.868 | -1.965 | <.001*** |
| iCloseness ~ Communication | 0.949 | 0.099 | 0.755 | 1.142 | <.001*** |
| sCloseness ~ Autism | 0.041 | 0.341 | -0.627 | 0.710 | .904 |
| sCloseness ~ Communication | 0.013 | 0.108 | -0.199 | 0.226 | .902 |
| Closeness0 ~ Maternal Distress0 | -0.048 | 0.038 | -0.122 | 0.026 | .207 |
| Closeness1 ~ Maternal Distress1 | -0.154 | 0.037 | -0.227 | -0.081 | <.001*** |
| Closeness2 ~ Maternal Distress2 | -0.157 | 0.040 | -0.236 | -0.079 | <.001*** |
| External0 ~ Maternal Distress0 | 0.082 | 0.023 | 0.037 | 0.128 | <.001*** |
| External1 ~ Maternal Distress1 | 0.129 | 0.025 | 0.080 | 0.178 | <.001*** |
| External2 ~ Maternal Distress2 | 0.154 | 0.025 | 0.106 | 0.203 | <0.001*** |
| Closeness0 ~ FEA0 | -0.216 | 0.111 | -0.432 | 0.001 | .051 |
| Closeness1 ~ FEA1 | 0.092 | 0.095 | -0.093 | 0.278 | .328 |
| Closeness2 ~ FEA2 | 0.113 | 0.115 | -0.112 | 0.339 | .324 |
| External0 ~ FEA0 | 0.139 | 0.068 | 0.005 | 0.273 | .042* |
| External1 ~ FEA1 | 0.047 | 0.063 | -0.077 | 0.171 | .460 |
| External2 ~ FEA2 | -0.093 | 0.072 | -0.235 | 0.049 | .198 |
| **Variances** | | | | | |
| iCloseness | 10.696 | 1.627 | 7.507 | 13.884 | <.001*** |

| | | | | | |
|---|---|---|---|---|---|
| sCloseness | 0.943 | 0.800 | -0.625 | 2.511 | .239 |
| iExternal | 4.745 | 0.721 | 3.332 | 6.157 | <.001*** |
| sExternal | 0.030 | 0.430 | -0.813 | 0.191 | .945 |
| Closeness0 | 7.163 | 1.509 | 4.205 | 10.121 | <.001*** |
| Closeness1 | 6.360 | 0.809 | 4.774 | 7.946 | <.001*** |
| Closeness2 | 7.202 | 1.654 | 3.960 | 10.444 | <.001*** |
| External0 | 2.894 | 0.658 | 1.605 | 4.184 | <.001*** |
| External 1 | 3.049 | 0.382 | 2.300 | 3.798 | <.001*** |
| External2 | 2.746 | 0.743 | 1.290 | 4.202 | <.001*** |
| **Covariances** | | | | | |
| Closeness ~~ iExternal | -2.163 | 0.621 | -3.381 | -0.945 | <.001*** |
| Closeness ~~ sExternal | -0.127 | 0.172 | -0.465 | 0.210 | .460 |

*Note.* ***$p$ = <0.001, **p=<0.01 *$p$= <0.05; s=slope factor; i=intercept factor; External=Child Externalising Behaviour Problems; Closeness = Mother-Child Closeness; CI = confidence intervals

**Table S12**

*Parameter Estimates for Conditional Parallel Process Growth Modelling of Trajectory of Mother-Child Closeness and Trajectory of Child Internalising Behaviour Problems*

| | Coefficient | Std. Error | CI (95%) Lower | CI (95%) Upper | *p* |
|---|---|---|---|---|---|
| **Intercepts** | | | | | |
| Closeness0 | 0.000 | | | | |
| Closeness1 | 0.000 | | | | |
| Closeness2 | 0.000 | | | | |
| Internal0 | 0.000 | | | | |
| Internal1 | 0.000 | | | | |
| Internal2 | 0.000 | | | | |
| iCloseness | 22.768 | 0.959 | 20.899 | 24.647 | <.001*** |
| iInternal | 2.085 | 0.776 | 0.564 | 3.605 | .007** |
| sCloseness | 0.626 | 1.898 | -3.094 | 4.346 | .741 |
| sInternal | 0.372 | 0.867 | -1.327 | 2.071 | .668 |
| **Regressions** | | | | | |
| sInternal ~ iCloseness | 0.028 | 0.032 | -0.035 | 0.091 | .383 |
| sInternal ~ iInternal | -0.163 | 0.052 | -0.265 | -0.061 | .002** |
| sCloseness ~ iInternal | 0.017 | 0.049 | -0.079 | 0.113 | .729 |
| sCloseness ~ iCloseness | -0.040 | 0.078 | -0.193 | 0.112 | .602 |
| iInternal ~ Autism | 3.325 | 0.395 | 2.552 | 4.098 | <.001*** |
| iInternal ~ Communication | 0.350 | 0.080 | 0.193 | 0.507 | <.001*** |
| sInternal ~ Autism | 0.465 | 0.241 | -0.008 | 0.938 | .054 |
| sInternal ~ Communication | 0.035 | 0.055 | -0.072 | 0.143 | .516 |
| iCloseness ~ Autism | -2.911 | 0.486 | -3.864 | -1.958 | <.001*** |

| | | | | | |
|---|---|---|---|---|---|
| iCloseness ~ Communication | 0.945 | 0.099 | 0.751 | 1.139 | <.001*** |
| sCloseness ~ Autism | -0.031 | 0.326 | -0.670 | 0.609 | .925 |
| sCloseness ~ Communication | 0.062 | 0.097 | -0.127 | 0.252 | .520 |
| Closeness0 ~ Maternal Distress0 | -0.043 | 0.038 | -0.116 | 0.031 | .258 |
| Closeness1 ~ Maternal Distress1 | -0.150 | 0.038 | -0.223 | -0.076 | <.001*** |
| Closeness2 ~ Maternal Distress2 | -0.150 | 0.040 | -0.228 | -0.072 | <.001*** |
| Internal0 ~ Maternal Distress0 | 0.092 | 0.030 | 0.033 | 0.150 | .002** |
| Internal1 ~ Maternal Distress1 | 0.145 | 0.030 | 0.087 | 0.204 | <.001*** |
| Internal2 ~ Maternal Distress2 | 0.102 | 0.030 | 0.042 | 0.161 | .001*** |
| Closeness0 ~ FEA0 | -0.200 | 0.110 | -0.417 | 0.016 | .070 |
| Closeness1 ~ FEA1 | 0.102 | 0.095 | -0.085 | 0.290 | .284 |
| Closeness2 ~ FEA2 | 0.111 | 0.115 | 0.337 | 0.111 | .336 |
| Internal0 ~ FEA0 | 0.370 | 0.088 | 0.197 | 0.542 | <.001*** |
| Internal1 ~ FEA1 | 0.233 | 0.075 | 0.085 | 0.381 | .002** |
| Internal2 ~ FEA2 | 0.217 | 0.087 | 0.046 | 0.388 | .013* |
| **Variances** | | | | | |
| iCloseness | 11.381 | 1.700 | 8.049 | 14.714 | <.001*** |
| sCloseness | 1.395 | 0.727 | -0.031 | 2.820 | .055 |
| iInternal | 8.704 | 1.129 | 6.492 | 10.916 | <.001*** |
| sInternal | 1.148 | 0.341 | 0.480 | 1.815 | .001*** |

| | | | | | |
|---|---|---|---|---|---|
| Closeness0 | 6.195 | 1.503 | 3.249 | 9.141 | <.001*** |
| Closeness1 | 6.772 | 0.836 | 5.135 | 8.410 | <.001*** |
| Closeness2 | 6.316 | 1.652 | 3.078 | 9.554 | <.001*** |
| Internal0 | 2.448 | 0.916 | 0.653 | 4.243 | .008*** |
| Internal1 | 4.607 | 0.539 | 3.550 | 5.664 | <.001*** |
| Internal2 | 3.224 | 0.946 | 1.370 | 5.077 | .001*** |
| **Covariances** | | | | | |
| iCloseness ~~ iInternal | -2.317 | 0.768 | -3.822 | -0.813 | .003** |
| sCloseness ~~ sInternal | 0.235 | 0.174 | -0.106 | 0.576 | .177 |

*Note.* ****p* = <0.001, ***p* = <0.01; **p*= <0.05; s=slope factor; i=intercept factor; Internal=Child Internalising Behaviour Problems; Closeness = Mother-Child Closeness; CI = confidence intervals

**Table S13**

*Parameter Estimates for Conditional Parallel Process Growth Modelling of Trajectory of Mother-Child Closeness and Trajectory of Child Prosocial Behaviour*

| | Coefficient | Std. Error | CI (95%) Lower | Upper | *p* |
|---|---|---|---|---|---|
| **Intercepts** | | | | | |
| Closeness0 | 0.000 | | | | |
| Closeness1 | 0.000 | | | | |
| Closeness2 | 0.000 | | | | |
| Prosocial0 | 0.000 | | | | |
| Prosocial1 | 0.000 | | | | |
| Prosocial2 | 0.000 | | | | |
| iCloseness | 22.884 | 0.955 | 21.012 | 24.755 | <.001*** |
| iProsocial | 1.652 | 0.507 | 0.660 | 2.645 | .001*** |
| sCloseness | -1.598 | 3.029 | -7.534 | 4.338 | .598 |
| sProsocial | 1.638 | 1.027 | -0.375 | 3.650 | .111 |
| **Regressions** | | | | | |
| sProsocial ~ iCloseness | -0.081 | 0.050 | -0.178 | 0.016 | .103 |
| sProsocial~ iProsocial | -0.009 | 0.109 | -0.222 | 0.205 | .936 |
| sCloseness ~ iProsocial | -0.333 | 0.200 | -0.726 | 0.059 | .096 |
| sCloseness ~ iCloseness | 0.080 | 0.142 | -0.198 | 0.359 | .571 |
| iProsocial ~ Autism | -1.337 | 0.260 | -1.846 | -0.828 | <.001*** |
| iProsocial ~ Communication | 0.585 | 0.053 | 0.482 | 0.688 | <.001*** |
| sProsocial ~ Autism | -0.411 | 0.145 | -0.695 | -0.128 | .004** |
| sProsocial~ Communication | 0.097 | 0.040 | 0.019 | 0.175 | .014* |
| iCloseness ~ Autism | -2.878 | 0.487 | -3.833 | -1.924 | <.001*** |

| | | | | | |
|---|---|---|---|---|---|
| iCloseness ~ Communication | 0.941 | 0.099 | 0.747 | 1.135 | <.001*** |
| sCloseness ~ Autism | -0.069 | 0.331 | -0.718 | 0.579 | .834 |
| sCloseness ~ Communication | 0.149 | 0.077 | -0.001 | 0.299 | .052 |
| Closeness0 ~ Maternal Distress0 | -0.039 | 0.038 | -0.113 | 0.035 | .299 |
| Closeness1 ~ Maternal Distress1 | -0.152 | 0.037 | -0.225 | -0.079 | <.001*** |
| Closeness2 ~ Maternal Distress2 | -0.155 | 0.040 | -0.233 | -0.077 | <.001*** |
| Prosocial0 ~ Maternal Distress0 | -0.046 | 0.020 | -0.085 | -0.007 | .019* |
| Prosocial1 ~ Maternal Distress1 | -0.046 | 0.019 | -0.083 | -0.009 | .016* |
| Prosocial2 ~ Maternal Distress2 | -0.063 | 0.020 | -0.101 | -0.024 | .001*** |
| Closeness0 ~ FEA0 | -0.230 | 0.109 | -0.444 | -0.016 | .036* |
| Closeness1 ~ FEA1 | 0.095 | 0.094 | -0.090 | 0.279 | .314 |
| Closeness2 ~ FEA2 | 0.119 | 0.114 | -0.105 | 0.343 | .299 |
| Prosocial0 ~ FEA0 | -0.148 | 0.057 | -0.261 | -0.036 | .010** |
| Prosocial1 ~ FEA1 | -0.015 | 0.048 | -0.108 | 0.079 | .016* |
| Prosocial2 ~ FEA2 | 0.093 | 0.056 | -0.017 | 0.203 | .098 |
| **Variances** | | | | | |
| iCloseness | 11.478 | 1.623 | 8.297 | 14.659 | <.001*** |
| sCloseness | 1.132 | 0.832 | -0.499 | 2.763 | .174 |
| iProsocial | 3.494 | 0.449 | 2.613 | 4.374 | <.001*** |
| sProsocial | 0.414 | 0.179 | 0.064 | 0.764 | .020** |
| Closeness0 | 6.207 | 1.352 | 3.557 | 8.857 | <.001*** |

| | | | | | |
|---|---|---|---|---|---|
| Closeness1 | 6.682 | 0.782 | 5.149 | 8.215 | <.001*** |
| Closeness2 | 6.533 | 1.516 | 3.562 | 9.505 | <.001*** |
| Prosocial0 | 1.450 | 0.352 | 0.760 | 2.140 | <.001*** |
| Prosocial1 | 1.751 | 0.204 | 1.351 | 2.152 | <.001*** |
| Prosocial2 | 1.293 | 0.371 | 0.567 | 2.020 | <.001*** |
| **Covariances** | | | | | |
| iCloseness ~~ iProsocial | 4.321 | 0.552 | 3.238 | 5.403 | <.001*** |
| sCloseness ~~ sProsocial | 0.754 | 0.152 | 0.456 | 1.053 | <.001*** |

*Note.* ****p* = <0.001, ** *p* = <0.01, **p*= <0.05; s=slope factor; i=intercept factor; Prosocial=Child Prosocial Behaviour; Closeness = Mother-Child Closeness; CI = confidence interval
